# Biomaterials for Dental Applications

**DOI:** 10.1155/2017/2520536

**Published:** 2017-03-15

**Authors:** Elif Bahar Tuna, Yoshiki Oshida, Bugra Ozen, Elizabeta Gjorgievska, Tamer Tuzuner

**Affiliations:** ^1^Faculty of Dentistry, Department of Pediatric Dentistry, Istanbul University, Istanbul, Turkey; ^2^Indiana University School of Dentistry, Indianapolis, IN, USA; ^3^University of California San Francisco School of Dentistry, San Francisco, CA, USA; ^4^Faculty of Dentistry, Department of Pediatric Dentistry, Istanbul Kemerburgaz University, Istanbul, Turkey; ^5^Department of Cardiology, Endodontology and Pedodontology, ACTA, University of Amsterdam and VU University Amsterdam, Amsterdam, Netherlands; ^6^Faculty of Dentistry, University “Ss. Cyril and Methodius” Skopje, Vodnjanska 17, 1000 Skopje, Macedonia; ^7^Faculty of Dentistry, Department of Pediatric Dentistry, Karadeniz Technical University, Trabzon, Turkey

With contributions, editorial team members in this special issue hold great insight. For this special issue, we received articles more than we expected from all over the world and ended up with 20 original research articles. With these articles, this issue offers comprehensive knowledge on biomaterials which serve a main role in the engineering of functional tissue replacements, both as supports for cell adhesion, vehicles for cell transplantation, and systems for controlled drug delivery and also provides studies on the application of the novel biomaterials in dentistry. Authors focused mainly on dental biomaterials especially with respect to their basic biological and chemical properties as well as clinical applications.

The development of new biocompatible materials and/or existing material composition and progressing techniques is expected to broaden the diversity of applications of biomaterials in dentistry field in upcoming years [1]. The progress in materials research including dentin bondings, impression materials, luting cements, glass ionomers, glass carbomers, composites, and ceramics clearly requires an improved understanding in multiple disciplines, as well as the development of new design methodologies in order to obtain better properties in biologic performance and better biocompatibility [2]. The objectives of all these biomaterials and technologies not only are to replace missing or damaged tooth tissues but are also now to promote tissue regeneration and also prevent healthy tooth tissue.

Comprehension of recent advances in biomaterial of dentistry would lead to appropriate applications of these biomaterials in clinical cases and successful strategies to improve dental treatment outcomes to better serve patients [3]. It is important to choose most appropriate material for the regeneration of the tooth structure via biomimetic processes. In other words, to choose the appropriate dental materials and its successful clinical use directly affect treatment outcome and long term results.

The biomaterials and technologies are not only replacing missing or damaged tissues but also promoting the tissue regeneration [4, 5]. There are many areas of research that biomaterials and dental stem cells were used. They offer potential for tissue regeneration in dentin, periodontal ligament, dental pulp, and even enamel. Also the use of dental stem cells as sources of cells to facilitate repair of nondental tissues such as bone and nerves has been introduced [5, 6]. In this issue, some researches related to MTA or Biodentine were accepted which have capability to stimulate tertiary dentin formation. However, as the cellular mechanisms behind successful tertiary dentin formation are largely unknown, few materials have been rationally designed to induce regeneration of root-like structures. Therefore, the editorial team thinks that these published articles can help the studies for explanation of unravelling cellular mechanisms behind dentin formation by pulp-derived stem cells, and, by this way, some other researcher may apply this knowledge to design novel biomaterials aimed at stimulating dentin formation by pulp-derived stem cells.

In this special issue, new topics have described the pediatric drugs on the color stability of dental materials, in vitro studies about composites, glass carbomer and calcium silicate cements, and some biomechanic research about oral implants. Besides, regarding white spot lesions and their treatment using with CPP-ACP paste, CAD/CAM systems and laser applications topics have been discussed with original articles.

In conclusion, we are now in the new era of biomaterials and biomaterial engineering including regenerative medicine, and applications are numerous in the modern dentistry. Advanced biomaterials inspired by biological systems and processes are likely to influence the development of novel technologies for a wide variety of applications. On the basis of these considerations, new biomaterials and technologies are playing the key role in the development of modern dentistry, and their development requires a multidisciplinary-based high quality researches. Comprehension of recent advances in biomaterial of dentistry would lead to finding the best application and the most successful treatment strategies to improve treatment outcomes of patients. The guest editorial team hopes that the present special issue provides for this aim and wish that this exciting topic would encourage other researchers for their future works.

*Elif Bahar Tuna* *Yoshiki Oshida* *Bugra Ozen* *Elizabeta Gjorgievska* *Tamer Tuzuner*
